# Aerosol-jet-printed, conformable microfluidic force sensors

**DOI:** 10.1016/j.xcrp.2021.100386

**Published:** 2021-04-21

**Authors:** Qingshen Jing, Alizée Pace, Liam Ives, Anke Husmann, Nordin Ćatić, Vikas Khanduja, Jehangir Cama, Sohini Kar-Narayan

**Affiliations:** 1 Department of Materials Science & Metallurgy, University of Cambridge, 27 Charles Babbage Road, Cambridge CB3 0FS, UK; 2 Cambridge Young Adult Hip Service, Addenbrooke’s-Cambridge University Hospitals, Box 37, Hills Road, Cambridge CB2 0QQ, UK; 3 Cavendish Laboratory, University of Cambridge, JJ Thomson Avenue, Cambridge CB3 0HE, UK; 4 Living Systems Institute, University of Exeter, Stocker Road, Exeter EX4 4QD, UK; 5 College of Engineering, Mathematics and Physical Sciences, Harrison Building, University of Exeter, North Park Road, Exeter EX4 4QF, UK

**Keywords:** conformable force sensor, aerosol-jet printing, microfluidics, capacitance, interdigitated electrodes, flexible electronics, soft robotics

## Abstract

Force sensors that are thin, low-cost, flexible, and compatible with commercial microelectronic chips are of great interest for use in biomedical sensing, precision surgery, and robotics. By leveraging a combination of microfluidics and capacitive sensing, we develop a thin, flexible force sensor that is conformable and robust. The sensor consists of a partially filled microfluidic channel made from a deformable material, with the channel overlaying a series of interdigitated electrodes coated with a thin, insulating polymer layer. When a force is applied to the microfluidic channel reservoir, the fluid is displaced along the channel over the electrodes, thus inducing a capacitance change proportional to the applied force. The microfluidic molds themselves are made of low-cost sacrificial materials deposited via aerosol-jet printing, which is also used to print the electrode layer. We envisage a large range of industrial and biomedical applications for this force sensor.

## Highlights

Highly flexible, capacitive force sensors based on microfluidics
Linear capacitance response to applied force
Rapid prototyping of low-cost and robust force sensors via aerosol-jet printing
Tunable design for various sensitivities and force measurement ranges

## Graphical abstract

**Figure:**
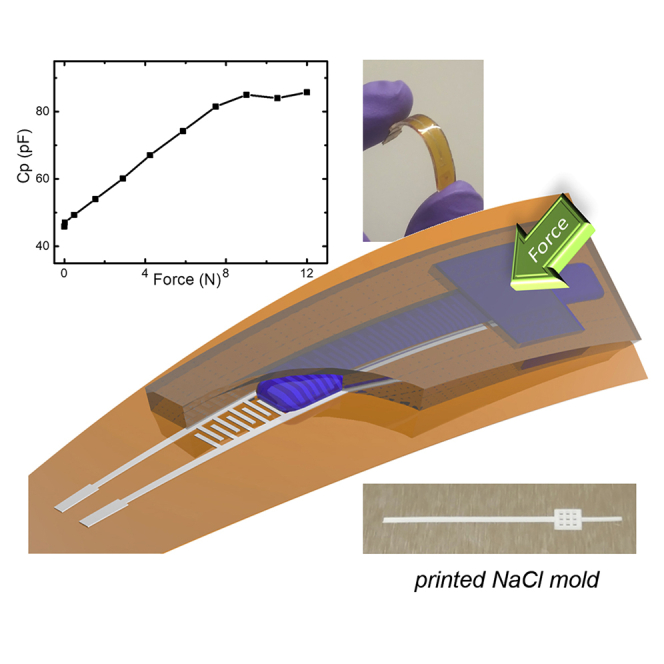

## Teaser

Jing et al. report a thin, conformable force sensor based on a combination of microfluidics and capacitive sensing. The low-cost sensor is fabricated via aerosol-jet printing and shows a highly linear response with adjustable design parameters for tunable sensitivities and force ranges.

## Introduction

Force-sensing requirements are ubiquitous across the fields of biomedical engineering, robotic surgery, and health monitoring, among others.^1^, ^2^, ^3^, ^4^, ^5^ The ability to provide real-time force monitoring can enhance the outcome of many surgical procedures. For example, force sensors are used to provide ligament-balancing information in knee-joint replacement surgery.^6^ They have also been deployed in the form of flexible capacitive sensors used to monitor the actual applied-force point during chest compression for cardiopulmonary resuscitation.^7^ Pressure sensors have also been reported for use in respiratory and pulse monitoring.^8^ Moving beyond the capabilities of more traditional force sensors, features such as flexibility, biocompatibility, ease of sterilization, functional integrity upon miniaturization, and a high degree of sensitivity are usually required in these biomedical or surgical applications, with solutions largely relying on nascent technologies and fabrication methods.

A wide range of techniques has been studied for force sensing, based on resistive, capacitive, magnetic, optical, piezoresistive, and piezoelectric-detection modalities,^9^, ^10^, ^11^, ^12^, ^13^, ^14^ with their general merits and demerits summarized by Dahiya et al.^15^ Traditional designs involving magnetic or optical elements and their detection parts are usually bulky in volume and sub-optimal for connecting to electrical circuitry.^16^ Resistive and optical sensors involve significant power consumption,^17^ whereas highly responsive piezoelectric materials are limited in material selection and thus restricted when it comes to biocompatible applications.^18^^,^^19^ Recent studies have also revealed the potential of leveraging triboelectric effects in force sensing,^20^, ^21^, ^22^, ^23^ with benefits including cost efficiency, self-powering capacities, and light-weight materials. However, the technology is mostly suited for detecting pulse forces or periodically dynamic forces, which can, thus, complicate monitoring of stable forces. In comparison, capacitive sensors, because of their low power consumption, simple structure, and highly sensitive response to deformation,^1^ are ideal candidates to form the basis of next-generation force sensors.

Typical capacitive force sensors contain parallel-plate electrodes, in which the distance between the plates and, hence, the measured capacitance, changes when an external load is applied.^24^ One drawback to this method is that the measurement of capacitance is non-trivial when the electrode area is reduced for local force detection because the capacitance value is proportional to the area of the electrodes.^25^ In addition, the capacitance change is often found to be non-linear as a function of the forces applied, which causes variations in the sensitivity based on the level of applied force and difficulties in sensor calibration.^10^ Patterned microfluidic devices,^26^ which possesses the benefits of miniaturization, cost efficiency, and scalability, offer a potential route to overcoming the challenges of traditional capacitive sensors. In this regard, a microfluidic-based tactile sensor containing a single-plate capacitor made from a pair of straight electrodes has shown good linear response with high sensitivity, based on the interfacial capacitance between the electrodes and an ionic liquid.^27^ However, the choice of usable ionic liquids is limited, and the micro-fabrication processes required for the development of such microfluidic devices are commonly based on complex lithography, which is not cost effective for scalability and is often time consuming when prototyping complex geometries.^28^^,^^29^

We have, instead, developed an all-printed, microfluidic-based force sensor that takes advantage of a state-of-the-art aerosol jet printer (AJP) and that can operate using a simple mixture of de-ionized (DI) water and glycerol. The process of AJP involves a programmable, adjustable injection of air-focused aerosols that contain target-material particles, which are generated via pneumatic or ultrasonic approaches onto desired substrates.^30^, ^31^, ^32^ More recently, this technology has been tested in making functional microfluidic devices.^33^ In the novel sensor design discussed in this article, instead of using parallel-plate electrodes adopted in previous works^10^^,^^25^, single-plate, interdigitated silver electrodes were directly printed to enhance the sensitivity of capacitance measurements. The adoption of a single-plate, interdigitated capacitor, a design which has been widely used for capacitance amplification,^34^^,^^35^ boosted the capacitance in a plate area that was limited by the size of the microfluidic channel. It also eliminated the “moving” electrodes, which were otherwise required in parallel-plate structured sensors, and successfully replaced the complex electrode assembly with a single printing step. Extremely cost-effective NaCl was printed as a water-soluble mold for a microfluidic channel and reservoir, followed with the casting of the microfluidic chip using polydimethylsiloxane (PDMS). The microfluidic chip was attached to a substrate with the printed interdigitated electrodes. The microfluidic channel was aligned with the electrodes, such that displacement of a fluid within the channel gave rise to a change in the capacitance between the electrodes. A liquid mixture containing DI water and glycerol was used in the device for a balance between non-volatility and sensor sensitivity. An external applied force drives the liquid from the reservoir of the microfluidic device into the channel, where it is detected by means of a capacitance change as the liquid coverage over the electrode increases. The range of measured forces may easily be adjusted by changing the dimensions of the microfluidic channel to adapt it to a wide range of applications. In this work, we specifically chose materials to guarantee the conformability of the sensor, which can be used under bending conditions or on curved surfaces. We quantified the effects of changing the electrode morphology, liquid permittivity, sensor thickness and geometry, as well as the thickness of the insulation layer over the electrodes, on sensor range, and on sensitivity, using a combination of experiments and finite-element analysis simulations. We demonstrated the feasibility of using the sensor for real-time feedback and “smart” control by using the sensor, attached to a curved surface, to control a robotic clamp. The conformable force sensors developed here are thin, flexible, and facilitate easy signal processing. The fabrication processes are low cost and are amenable to fast prototyping and mass manufacturing. We thus demonstrate the development and characterization of a novel, conformable, thin, and low-cost force sensor, with potential use in a range of biomedical, engineering, and robotic applications.

## Results and discussion

### Structural design

**Figure 1:**
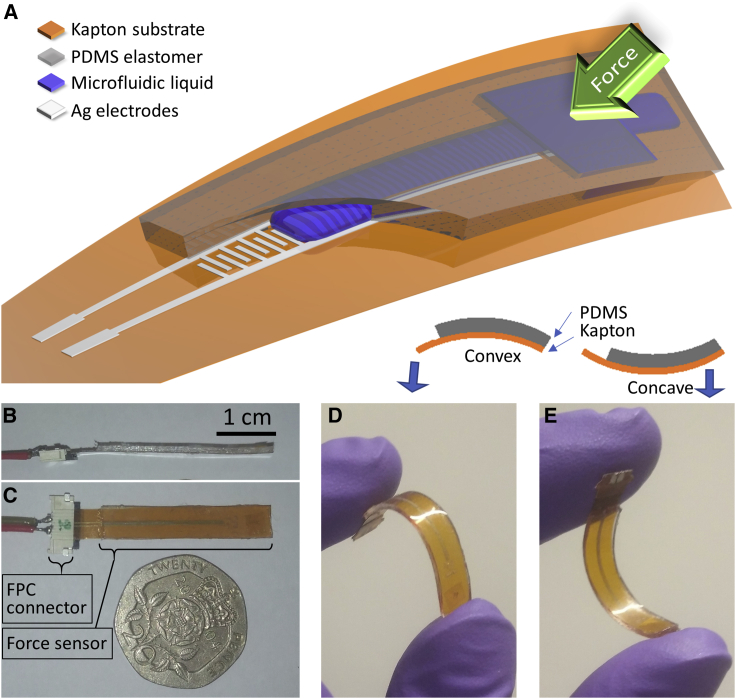
Microfluidics-based force sensor (A) Schematic of the novel microfluidics-based force sensor showing its components: a PDMS microfluidic chip containing a microfluidic channel, a reservoir, and an injection channel, aligned and bonded on top of interdigitated silver electrodes, which are themselves printed on a flexible Kapton substrate. The reservoir is filled with a solution of glycerol and water (2:1 ratio by volume). On application of an external force, the fluid is displaced along the channel and passes over the electrodes, inducing a change in capacitance. (B and C) Side (B) and top (C) view of the fabricated force sensor. FPC, flexible printed circuit. (D and E) The sensor can be flexibly bent in either a convex (D) or a concave (E) manner.

The microfluidic force sensor was composed of a flexible polyimide (PI, Kapton) substrate, a pair of interdigitated electrodes, and a microfluidic chip (PDMS) consisting of a single channel (20 mm × 0.5 mm × 0.2 mm, length [L] × width [W] × height [H]) with a reservoir (2 mm × 2 mm × 0.3 mm, L × W × H) toward one end. The PDMS chip was aligned over the electrodes and bonded to the Kapton to form the microfluidic chamber with the end of the channel opposite to the injection side open to air; the device schematic is shown in Figure 1A. The surface area on top of the reservoir was the active sensing area, and the reservoir itself was filled with a dielectric liquid containing a mixture of glycerol and DI water at a 2:1 volume ratio. Upon application of an external load to the sensing area, the dielectric liquid was displaced along the microfluidic channel because of the mechanical deformation of the reservoir caused by the external force, which, in turn, changed the measured capacitance between the interdigitated electrodes printed on the Kapton substrate beneath the channel, based on the coverage of the electrodes by the displaced liquid. Removing the external force enabled the recovery of the reservoir to its original shape because of the elastic property of PDMS, and the corresponding retraction of the liquid front in the channel restored the capacitance to its original value. The change of the measured capacitance was, therefore, found to be proportional to the force applied to the sensing area. The sensor had dimensions of 5 mm in width, ∼1 mm in thickness, and approximately 3 cm in length (Figures 1B and 1C) and could be freely bent into either convex or concave shapes because of the flexibility of the materials it was made from (Figures 1D and 1E).

### Device fabrication

**Figure 2:**
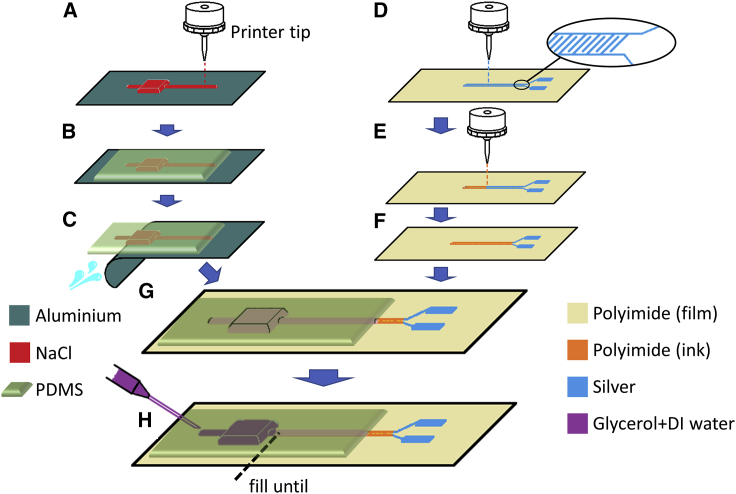
Schematic of the sensor fabrication process (A) The NaCl mold is directly deposited on an aluminum substrate via aerosol jet printing. (B) PDMS is cast over the NaCl mold after printing and cured. (C) After the PDMS is cured, the aluminum substrate is torn off, and the NaCl washed with DI water. (D) Direct aerosol jet printing of Ag interdigitated electrodes on a PI (Kapton) substrate. The inset shows the enlarged electrode pattern. (E) Aerosol-jet printing of the PI insulation layer on top of the electrodes. (F) The printed Kapton only covers the interdigitated electrodes, not the connectors. (G) The PDMS chip and electrode hosting Kapton substrate are aligned and bonded together with glues (for details see Table S1). (H) The sensor liquid, a mixture of glycerol and DI water at a 2:1 volume ratio, is injected into the microfluidic device via the shorter channel behind the reservoir.

**Figure 3:**
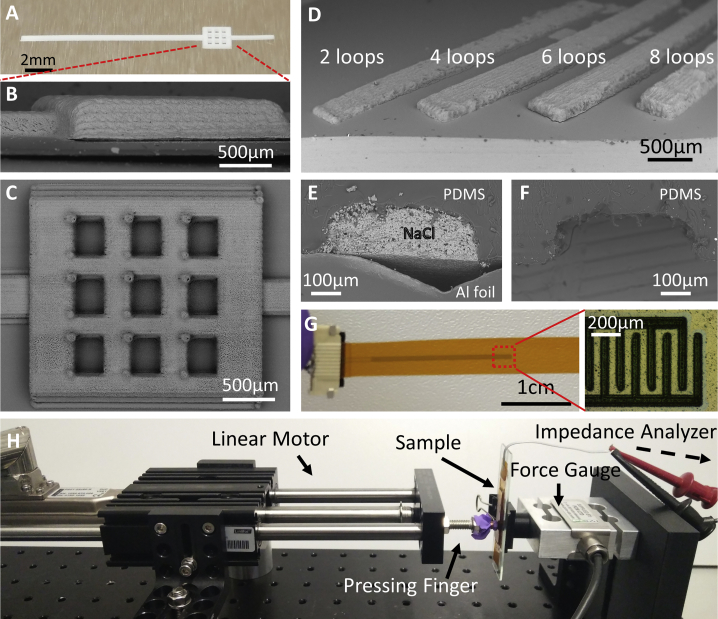
Channel mold, electrodes, and measurement setup (A) Photograph of the printed NaCl mold. (B) SEM side view image of the reservoir in the mold. (C) SEM top view image of the mold reservoir showing the 3 × 3 matrix of wells designed to prevent the reservoir of the PDMS replica chip from collapsing under external load. (D) NaCl molds of various thicknesses obtained by repeatedly printing a design using multiple printing loops. (E) SEM cross-sectional view of an NaCl mold embedded in PDMS. (F) SEM cross-sectional view of a PDMS microfluidic channel after the removal of NaCl and the Al foil. (G) Photo of the electrode printed on a PI base, with the inset showing the detailed view of the interdigitated electrodes obtained by optical microscopy. (H) Compression testing rig built from a linear motor, a pressing finger (moving part), a force gauge (fixed part), and an impedance analyzer. The sensor was mounted on the force gauge with the sensing area facing the pressing finger, which was, in turn, driven by the linear motor.

The fabrication processes of the microfluidic force sensor involved the following steps, as schematically depicted in Figure 2: microfluidic channel fabrication, electrode fabrication, bonding, and liquid injection. The mold for the microfluidic channels was printed using an aerosol-jet printer,^33^ with the NaCl “ink” deposited on an aluminum film (Figure 2A). The mold design included a reservoir connected to a long, straight channel and a short channel for fluid injection on opposite sides of the reservoir. The square reservoir has dimensions of 2 mm × 2 mm × 0.3 mm (L × W × H), and the long, straight channel has dimensions of 2 cm × 0.5 mm × 0.2 mm (L × W × H). To prevent the reservoir of the PDMS device from collapsing and to provide better structural stability during the application of repeated cycles of external force, 9 square, hollow wells, aligned in a 3 × 3 matrix, were incorporated into the design of the reservoir (Figure 3C); these formed additional supports within the reservoir during PDMS casting. Liquid PDMS was then poured on top of the mold and cured (Figure 2C), followed by the removal of the aluminum film, which was simply torn off, and the subsequent removal of the NaCl by washing with DI water (Figure 2C).

The Ag interdigitated electrodes were directly printed with the aerosol-jet printer on 75-μm-thick Kapton (PI) films (Figure 2D); on top of which, a PI layer of the same pattern was printed to completely cover the interdigitated area (Figure 2E). This coating served to protect the electrodes from direct contact with the microfluidic liquid and also added robustness during assembly and bending. By using a wider printer tip (300-μm tip, compared with the 150-μm tip used for electrode printing; see Experimental procedures section) and because of the slight overflow of the PI ink during the printing, the area of the Ag electrodes was fully covered by PI, with the exception of the ends designed for electrical connections (Figure 2F).

The PDMS chip was then attached on top of the PI film using a primer and a silicone glue (see Experimental procedures section) to obtain a good seal. We tested a range of different glues for this purpose, and the results are summarized in Table S1. The long, microfluidic channel was fully aligned with the interdigitated electrodes under a microscope (Figure 2G), leaving the far end of the channel (opposite to the reservoir) open to air. The device was then tailored into a suitable width using a scalpel. This was followed by the injection of a liquid mixture of DI water and glycerol from the shorter channel until the reservoir was fully filled (Figure 2H). We chose this liquid mixture^36^ to balance the volatility of the water and the relatively low permittivity of pure glycerol (as compared with pure water). The injection hole was then sealed with Kapton (PI) adhesive tape. Electrical contact for capacitance measurements was made to the electrodes using a flexible printed circuit (FPC) connector, by tailoring the ends of the PI substrate and electrodes into a shape amenable for direct clamping by the connector. The capacitance value of the interdigitated electrodes was then monitored via an impedance analyzer.

### Mold and channel characterization

A photograph of the printed NaCl mold on the Al foil is presented in Figure 3A, with a detailed side (Figure 3B) and a top (Figure 3C) view obtained by scanning electron microscopy (SEM). The 9 square, hollow wells, whose purpose was to prevent the collapse of the reservoir under external load, were fabricated by AJP (Figure 3C). To build up the thickness, each mold was printed with multiple loops of 3 different patterns (Figure S1). A loop in AJP is defined as the completion of a single run of an input printing pattern. The thicknesses of the mold were precisely controlled by the number of loops used to print the mold.^33^ Specifically, channel thicknesses varying between ∼50 μm and 200 μm were achieved by repeatedly printing patterns with 2–8 loops (Figure 3D), yielding a thickness of approximately 25 μm per loop under these conditions. A cross-sectional view of the mold when covered in PDMS was examined by SEM (Figure 3E), with the Al foil substrate still attached at the bottom for the print. For comparison, the cross-section of a segment of a PDMS microfluidic channel from the same piece was also imaged after the removal of the substrate and NaCl (Figure 3F). Some roughness on the inner side of the channel wall was observed because of the surface texture of the NaCl mold. This is dependent on the printing direction; the printing in this instance was patterned along the length of the channel. This level of roughness was expected to have little influence in restricting the fluid flow inside the channel. The quality of the interdigitated electrodes printed on the Kapton films (Figure 3G) was confirmed by visual examination using optical microscopy, with the pitch of the electrode measured at 200 μm (Figure 3G, inset).

### Sensor performance testing under external force

The performance of the sensors was tested by varying the force using a programmable motor that was set up with a force measurement system and an electrical measurement system (Figure 3H). The setup included a linear motor (LinMot) that provided a one-directional, propelling force over a small moving distance on a micrometer-length scale. A “pressing finger” that provided a circular contact area with a 5 mm diameter was attached to the end of the linear motor for accurately applying the force on the active sensing area of the sensor (i.e., the area containing the reservoir). The sensor was fixed to a glass slide, which was itself attached to a force gauge. Both the force applied on the sensor and the corresponding capacitance value from the sensor electrodes were simultaneously monitored through a controller and an impedance analyzer (model 4294A, Agilent Technologies) for data collection. We note that the sensor design is easily customizable, so, for objects of different sizes, the force-sensing area can be adjusted accordingly. Further, for much larger objects, an array of such sensors can be used.

**Figure 4:**
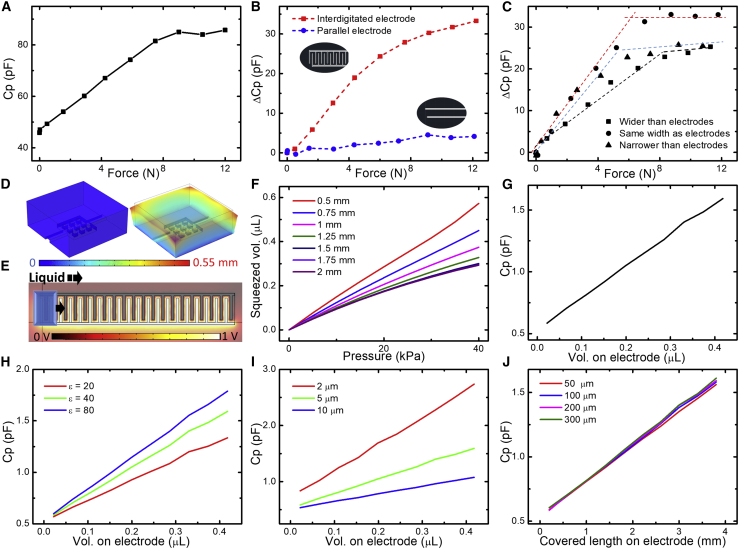
Performance of the force sensor and parameter study based on experiments and simulations (A) Capacitance change in response to force in a typical sensor. (B) Comparison of different electrode morphologies shows that interdigitated electrodes possess larger sensitivity than parallel electrodes. (C) Comparison of the capacitance response in sensors of various channel widths, including 3 different channels whose widths are either greater than (750 μm), the same (500 μm), or narrower than (250 μm) the width of the electrodes (500 μm). (D) Simulated displacement distribution of a 5 × 5 mm^2^ area of PDMS containing the reservoir before and after a force of 7 N is applied. (E) A demonstration showing how the capacitance between the electrodes is simulated in response to a growing column of liquid over the electrodes, when the setting is a 1 V potential bias to the electrodes. The blue object represents the liquid coverage that expands toward the right over the electrodes. (F) Simulation results of volume decreases of the reservoir under forces, based on different thicknesses of PDMS (varied from 0.5 to 2 mm). (G) Simulation results of capacitance versus volume of liquid on top of the electrode. Capacitance increases linearly with the length of electrodes covered by the liquid. (H) Simulation results comparing the sensitivity for liquids of different relative permittivity (ε, varied from 20 to 80). Higher ε yields larger sensitivity. (I) Simulation results presenting the effect on the capacitance sensitivity of varying the thickness of the PI insulation layers (varied from 2 to 10 μm) on top of the electrodes. (J) Simulation results showing that, given a certain length of liquid coverage, the thickness of the liquid (varied from 50 to 300 μm) on top of that area has little effect on the capacitance.

A typical force-versus-capacitance measurement from the sensor is shown in Figure 4A; in which, we observed that the corresponding relationship between the force applied and the corresponding capacitance could be divided into 2 distinct regimes. Up to an applied force of around 9 N, the sensor responded with an approximately linear change in capacitance, with a sensitivity of 4.3 pF/N. The result reflected the elastic deformation of the PDMS with the increase of the external force, which led to a decrease in the volume of the reservoir and, therefore, a corresponding change of the capacitance between the electrodes because of the displaced liquid moving into the channel. These hypotheses were further examined using finite-element modeling (FEM). For a force above 9 N, the capacitance change was negligible, possibly because of an almost-complete draining of the liquid volume in the reservoir or, alternatively, because the displaced liquid front reached the open end of the channel at the end of the interdigitated electrodes. We, therefore, consider the operating measurement range of the sensors to be the range of forces corresponding to this linear regime of external force versus measured capacitance change. That range can be enhanced by increasing the reservoir volume and by making adjustments to the channel length/height.

The hysteresis behavior of the sensor was also characterized by monitoring the capacitance from the sensor upon both compression and release; the different cycles showed almost identical capacitance-force curves (Figure S2). Constant forces were also applied on the sensor for up to 120 s with no drift in the capacitance observed within the monitoring window (Figure S3). The effect of temperature on the sensor output was also explored. Because the sensor relies on the motion of a liquid as the sensing mechanism, it is expected to only work for temperatures at which the material remains in liquid form (e.g., between freezing and boiling points). For the materials used in our sensor, we tested the temperature dependence by placing the sensor over a plate that was either at room temperature (20°C), cooled (10°C), or heated (35°C and 50°C). The response curve (Figure S4) showed a drift toward higher capacitance values when the temperature was increased (i.e., the capacitance simply started from a higher initial value when the temperature was increased). Importantly, however, the sensitivity (i.e., the gradient of the capacitance-force curve) remained similar across that temperature range. Given that neither the permittivity nor the thermal volume expansion of our chosen liquid is expected to change much within that temperature range,^37^ the drift was probably caused by the temperature dependence of capacitance^38^ between the interdigitated electrodes. Because the temperature changes (within our measured range) did not affect the sensitivity, the temperature effect can be accounted for with a simple initialization step, which may involve zeroing the capacitance at the zero-force initialization step when using the sensor.

The geometrical design of the electrodes and microfluidic channel were expected to have a significant influence on the sensitivity of the sensors. We compared the sensitivity between our interdigitated electrode design and a simple, straight, parallel-electrodes design (Figure 4B). The width of the parallel electrodes (distance between the electrodes) was 500 μm, the same as the width of the interdigitated electrodes (Figure S5); this was also similar to the width of the microfluidic channel itself. The sensitivities presented in Figure 4B show a huge difference between the 2 designs, with up to 3.75 pF/N acquired from our interdigitated electrode design, as compared with 0.55 pF/N acquired from the simple, parallel-electrodes design. Furthermore, we fixed the reservoir size and changed the width of the microfluidic channel to be either wider (750 μm), the same width (500 μm), or narrower (250 μm) than the width of the interdigitated electrodes (500 μm), which were maintained at a fixed size (Figures 4C and S6). The sensor with narrower microfluidic channels showed similar sensitivity to the one that had the same width at the initial part of the measurement range. However, the total force measurement range was smaller because of the liquid front reaching the open end of the channel under smaller forces because of the smaller volume of the channel. The wider channel, in contrast, showed a larger measurement range but with less sensitivity, as expected, because the volume of liquid covering a defined portion of the sensor was larger than in the other cases. The sensors tested for these experiments were fabricated from electrodes and microfluidic channels made under similar conditions to avoid any small changes in the manufacturing process having an effect on the comparative measurements.

### FEM simulation studies

A series of simulation studies were conducted by varying a number of parameters that could potentially affect the performance of the sensors. Two groups of distinct (yet related) simulations are shown. One investigated reservoir deformation in response to external force over PDMS (Figure 4D), whereas the other studied the functional dependence of the capacitance of the electrodes on the liquid volume covering the electrodes (Figure 4E). Simulations of sensor function in response to varying the thickness of the PDMS chip were also performed. Results in Figure 4F reveal that a thicker cast of PDMS leads to smaller-volume decreases of the reservoir under the same external force (as expected), resulting in an enhanced measurement range, although at the cost of requiring a thicker device and also a corresponding loss in sensitivity. The volume of the reservoir decreased approximately linearly under an external force for all PDMS thicknesses studied. Correspondingly, the same volume of liquid will be “squeezed” from the reservoir into the channel in response to the decreasing volumes of the reservoir. The capacitance measure increased linearly as the liquid covered the electrodes, when it was forced from the reservoir by the external force. This can be quantified by studying the effect of different volumes of liquid covering the length of the electrodes, and we obtained a linear relationship between capacitance and liquid volume from the simulation results (Figure 4G). An additional simulation showed that, once the reservoir was bent, the propelled volume of liquid was slightly smaller than that in the planar configuration with the same applied force (Figure S7). We envisage the sensor being used in curved geometries with a fixed curvature; for optimal use, the sensors should be calibrated after installation in the curved geometry.

### Robustness and flexibility demonstration

**Figure 5:**
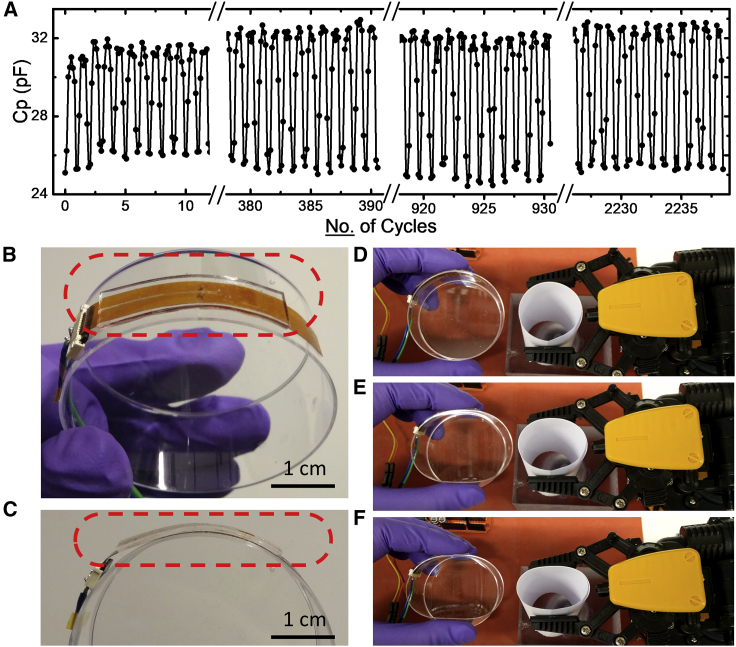
Stability test and robotic clamp interaction of the force sensor (A) Stability test of a single sensor over more than 2,200 cycles. The force sensor was pressed and released with a force of up to 7 N at a frequency of 0.1 Hz. (B and C) The force sensor was attached to the curved surface of a Petri dish. (D–F) The sensor monitored the force applied on the Petri dish from (D) no compression, (E) a medium compression, and (F) a hard compression, with the values collected and sent to command a robotic clamp, which mirrored the clamping mechanism accordingly.

To examine the stability of the sensor over multiple measurements, a fatigue test was conducted. The microfluidic sensor was continuously compressed and released at a frequency of 0.1 Hz under a force of up to 7 N. The responses remained stable for more than 2,200 cycles, as displayed in Figure 5A. To further test the performance of the sensors in curved geometries, the sensor was attached to the curved side wall of a deformable, plastic Petri dish (Figures 5B and 5C) as a controller and was used to detect the force applied when squeezing the Petri dish by hand. In response to the squeezing, a robotic claw was made to react by opening and closing its clamp to varying degrees based on the amplitude of the force applied to the sensor on the Petri dish. The capacitance values of the sensor were then measured and collected with a micro controller unit (MCU) that communicated with a computer, where the forces were calculated. Based on the force (or capacitance), commands were sent to the robotic claw to perform clamping and releasing motions in real time. Figure S8 shows a recorded capacitance value over time from the controller, where 4 thresholds were pre-set by the program to trigger actions of the clamp containing various opening/closing angles. Figures 5D–5F demonstrate the actions of the clamp according to a series of increasing forces applied on the Petri dish, with the complete performance recorded in Video S1.

Video S1. Demonstration for the force sensor in robotic control applications The sensor was attached to the outer circumference of the curved wall of a plastic Petri dish to detect the force the operator applied to squeeze the Petri dish. The robotic claw was programmed to mirror the operator’s action based on the force detected from the sensor.

We envisage that this sensor will be most resilient (in curved geometries) when it is bent and fixed in a particular configuration, as demonstrated in the experiment above. If the entire sensor were itself subjected to rough handling (continuous bending and flexing without due care), the materials making up the sensor may tear, or the liquid within the reservoir/channel may break into pockets, causing air bubbles and disruption to the capacitance (and hence force) measurements. Once the entire sensor is fixed in place, in either curved or planar geometries, the force-sensing capability is robust, as has been demonstrated throughout this article.

In conclusion, we have demonstrated the development of a novel, conformable, microfluidics-based force sensor made by AJP technologies. The microfluidic channel is made with PDMS-based soft lithography from a mold constructed by the direct printing of cheap, sacrificial NaCl materials. Importantly, the microfluidic channel is then aligned with interdigitated electrodes also printed directly on a polyimide substrate, and thus, the construction of the device in its entirety is facilitated by the AJP techniques we have previously developed.^33^ The sensor is compact and yet can provide force measurements up to 9 N with an approximately linear sensitivity of 3.75 pF/N. Importantly, the sensitivity and measurement range can be tuned by changing fabrication parameters, such as the width of the microfluidic channel, the size of the reservoir, the thickness of the PDMS layer, and the liquids (hence the permittivity) used. Our sensors are easily customizable in terms of the reservoir size and, hence, can be optimized to ensure that the contact area associated with the applied force matches the reservoir size, or alternatively, an array of sensors can be deployed to achieve spatial resolution of the applied force. The sensors are capable of enduring repeated cycles of measurement and can be attached to curved surfaces for force monitoring. We have demonstrated its use for remotely controlling a robotic clamp with a real-time force measurement and feedback mechanism. Our microfluidic force sensors are low cost, thin, conformable, amenable to fast prototyping, and can be scaled for mass production; they also show characteristics such as a linear response to external loads and convenient zeroing. The capacitance measurement could potentially be combined with remote approaches for maintenance-free scenarios. We envisage this conformable force sensor will find a range of commercial applications in robotics and biomechanical and biomedical engineering.

## Experimental Procedures

### Resource availability

#### Lead contact

Further information and requests for resources should be directed to and fulfilled by the lead contact, Sohini, Kar-Narayan (sk568@cam.ac.uk).

#### Materials availability

Commercially available reagents were used as described below in Fabrication. No new unique reagents were generated in this study.

#### Data and code availability

The authors declare that data supporting the findings of this study are available within the article, the Supplemental information, and the DSpace@Cambridge data repository (https://doi.org/10.17863/CAM.63758).

### Fabrication of the sensor

*Interdigitated electrodes:* 1.2-mL Ag nanoparticle ink (PRELECT TPS 50, Clariant) diluted with DI water at a 1:1 volume ratio was used with the ultrasonic atomizer installed in the AJP (Aerosol Jet 200, Optomec) with a tip size of 150 μm to print the electrodes on PI film (RS Components) at 75 μm thickness. The overall dimensions of the interdigitated electrodes were 2 cm × 0.5 mm (L × W). The end of the electrodes was designed and printed with connecting pads that fitted the required size of a 2-pin FPC connector with a 1-mm pitch. A 15-mL PI ink, made from a mix of poly(pyromellitic dianhydrideco-4,4′-oxydianiline), amic acid solution (12.8 wt% in 80% NMP/20% aromatic hydrocarbon, Sigma-Aldrich), and N-methyl-2-pyrrolidone (NMP, Sigma-Aldrich) at a 1:1 volume ratio, was used with the pneumatic atomizer and a tip size of 300 μm for printing the insulation layer. All the electrodes were covered with PI, except for the connecting pads that were needed for the FPC connectors.

*PDMS microfluidic chip:* 1.2-mL saturated NaCl (Sigma-Aldrich) water solvent was used with the ultrasonic atomizer to print the mold on Al film with a tip size of 300 μm. In total, 12 printing loops (4 loops for each pattern shown in Figure S1) were required to obtain the desired mold thickness. Printing was conducted as the Al film was heated up to 95°C by the plate stage beneath it, and thus, water in the NaCl “ink” directly evaporated during deposition. Liquid PDMS (DowSil Sylgard 184) was made by mixing the silicone elastomer and the crosslinking agent in a 10:1 weight ratio. The uncured PDMS was poured on top of the mold with the Al substrate positioned (flat) on a hot plate. The PDMS was then cured at 70°C for 1 h. After curing, the Al film was easily removed, and the NaCl was washed with DI water.

*Attachment of the PDMS microfluidic chip to the electrode substrate:* A thin layer of primer (DOWSIL 1200) was first applied on the surface of the Kapton film and left for 1 h until fully dried. A thin layer of silicon glue (DOWSIL 3140) was applied on top of the primer, followed by the attachment of the PDMS microfluidic chip.

*Injection of the liquid:* The mixture of glycerol and water (2:1 ratio) was injected with a syringe from the injection channel side. The injection was performed carefully to avoid the formation of any air bubbles. If, despite precautions, a bubble does form, a force can be applied at the sensor’s sensing area to push out extra liquid/bubbles from the open end of the channel and ensure that the device is filled appropriately.

### Measurements

A linear motor (LinMot) and a force gauge (AEP transducers) were mounted on the same platform, and a flat-head screw with its head wrapped with a nitrile rubber sheet was mounted onto the linear motor’s arm as the “pressing finger.” After the sensor was installed between the pressing finger and the force gauge (Figure 3H), the linear motor was set to move forward to compress the sensor at an increment of 100 μm/step. Forces read from the force gauge were recorded, and the capacitance of the electrodes was monitored by an impedance analyzer (model 4294A, Agilent Technologies) at a measuring frequency of 800 Hz. In the robustness test, the linear motor was set to conduct a reciprocating motion with a stroke of 1 mm and a frequency of 0.1 Hz. A maximum force of 7 N was recorded under such periodic testing.

### Simulations

Multiphysics (COMSOL) was adopted for the simulations reported in Figure 4. Mooney-Rivlin with 5 parameters was used in simulating reservoir deformation with the co-efficient acquired from experimental compression and tension data: C10 = −23,153 Pa, C01 = 242,123 Pa, C11 = 36,083 Pa, C20 = −7,760 Pa, C02 = −33,635 Pa, and bulk modulus 962 MPa. Sizes of the reservoir from the actual devices were applied in the simulations.

An electrostatic model was used for the capacitance simulations. The capacitances were calculated by setting 1 electrode to 1 V and the other to 0 V with other conditions being adjusted and studied as required. Sizes of the electrodes were from the actual devices but with the total length of the electrode reduced to 4 mm for time-efficient simulations (which is the main reason why the absolute values of capacitance differ between the experiments and simulations).

### Demonstrations

A microfluidic force sensor was bent convexly and attached by double-sided tape to the outer wall of a plastic Petri dish with a diameter of approximately 5 cm. An MCU (Orangepip Kona328, an Arduino Uno compatible development board, Rapid Electronics) was applied to collect capacitance values and transmit data to the computer via a universal serial bus (USB) cable for real-time feedback. The program running on the computer that collected data from the MCU and sent commands to the robotic claw (Robert Arm, distributed by FADISEL) via USB cable was written in Python. Capacitances at a minimum applied force (no force) and a maximum applied force were read from the sensors for calibration and initialization, with the acquired range normalized and evenly divided into 5 steps for 5 different levels of force detection and robot action.

## Supplemental information

Document S1. Figures S1–S8 and Table S1

Document S2. Article plus supplemental information
