# Supplementary material for: Aerosol-jet-printed, conformable microfluidic force sensors
Source: Cell Rep Phys Sci. 2021 Apr 21;2(4):100386. doi: 10.1016/j.xcrp.2021.100386 (PMC8063179; doi:10.1016/j.xcrp.2021.100386)

Cell Reports Physical Science, Volume *2*

Supplemental information

Aerosol-jet-printed, conformable microfluidic force sensors

Qingshen Jing, Alizée Pace, Liam Ives, Anke Husmann, Nordin Ćatić, Vikas Khanduja, Jehangir Cama, and Sohini Kar-Narayan

Table S1: Glue tests. Epoxy (Loctite 9483) and silicone-based glues (PDMS, Dowsil 3140, Dowsil 732) were tested for bonding the PDMS to the Kapton film. Bonding strength was examined based on whether or not there was liquid leakage from the device on the application of external forces. The results are summarized in the table below. Most adhesives were either good for Kapton or PDMS but not for both. We finally chose to use a primer (Dowsil 1200) on the surface of Kapton to increase the adhesion between Dowsil 3140 silicone glue and Kapton, which achieved a good sealing result.

Glue type	Attachment to Kapton	Attachment to PDMS	Note
Uncured PDMS (SYLGARD 184)	No	Yes	
Epoxy (Loctite 9483)	Yes	No	
DOWSIL 3140	No	Yes	
DOWSIL 732	Yes	Yes	Non-flowable but good strength
732 mixed with PDMS (Uncured)	Yes	Yes	Flowable with slightly reduced strength from 732
DOWSIL 1200 Primer + 3140	Yes	Yes	Strong bonding yet need two steps to apply the glues

Figure S1: AutoCAD designs of the NaCl mold. Each mold was printed with multiple layers of three different patterns, from the bottom to the top shown in this figure. Red lines represent the printing paths.

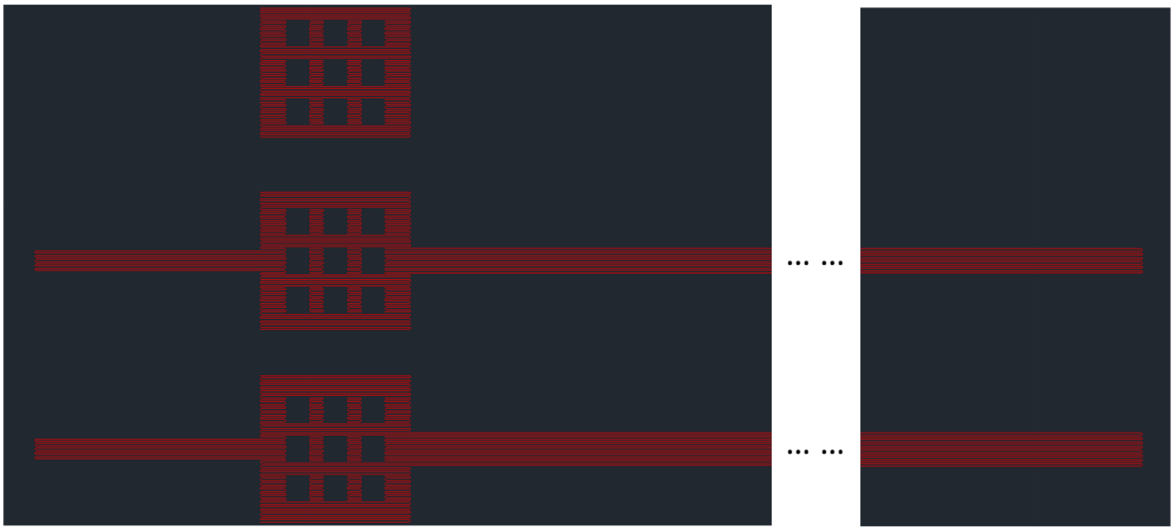

Figure S2: Hysteresis measurement of the capacitance from a representative force sensor when being compressed and released, for 2 continuous rounds. The combination of this measurement and stability demonstrated in section 2.6 together show negligible hysteresis effect of the sensor.

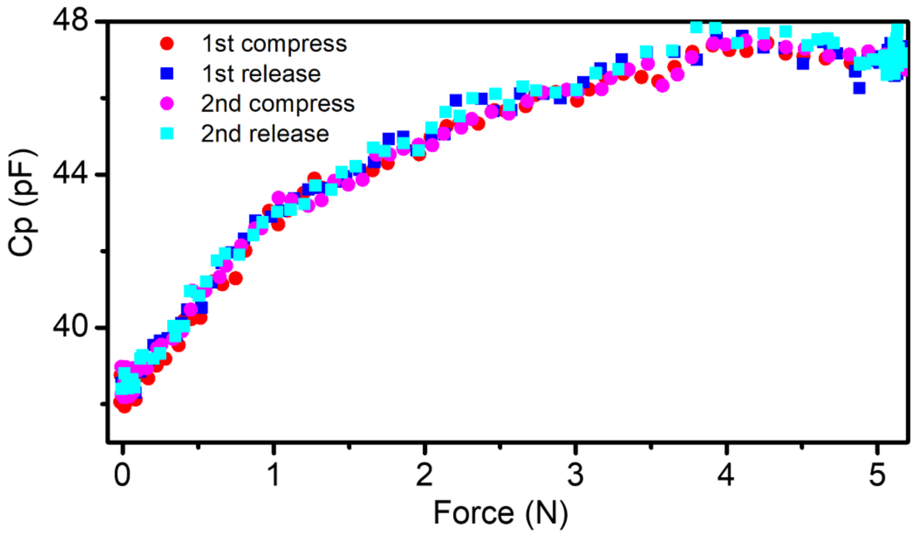

Figure S3: Capacitance values from the force sensor under continuous compression at several forces for up to 120 s. No drift in the capacitance was observed within these timescales.

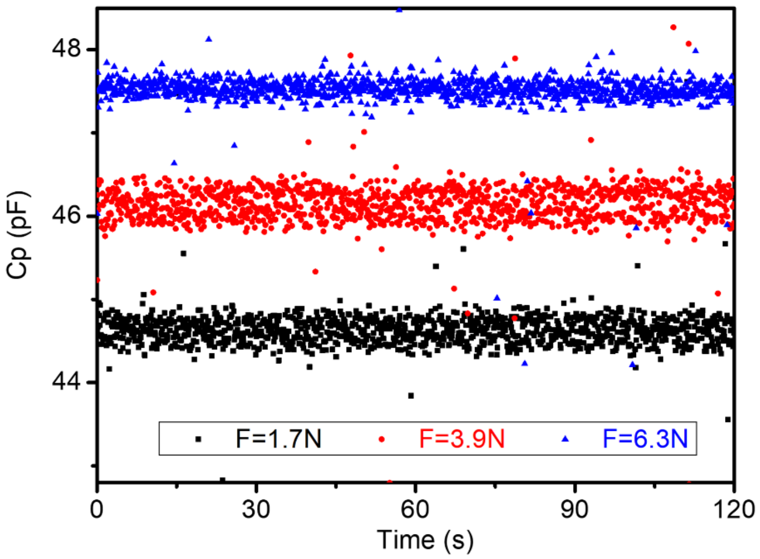

Figure S4: Temperature response of the force sensor. Results showed an increase in the initial value of the capacitance with increasing temperature. However, the sensitivity remains similar within this temperature range, and hence the temperature factor can be accounted for by simply zeroing out the shift in the absence of any applied force.

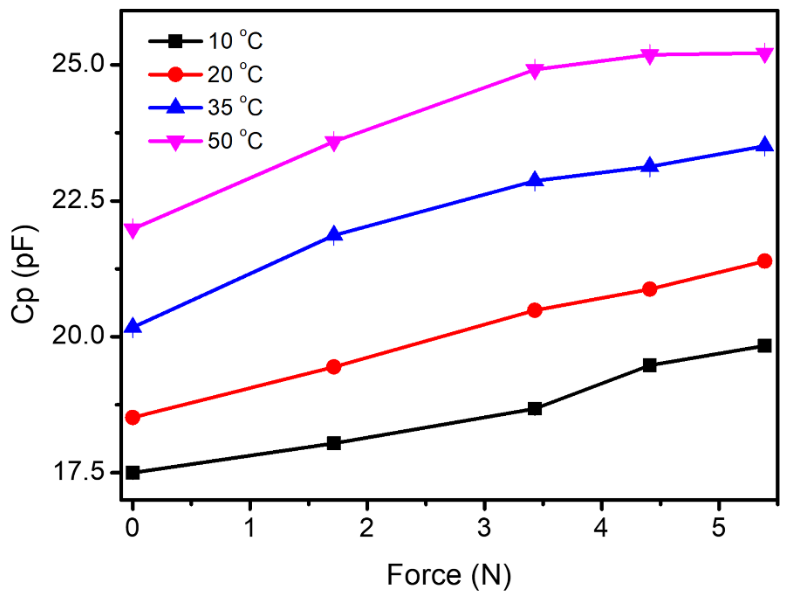

Figure S5: Illustrations of the comparison in design between parallel electrodes and interdigitated electrodes. The electrodes are shown in blue.

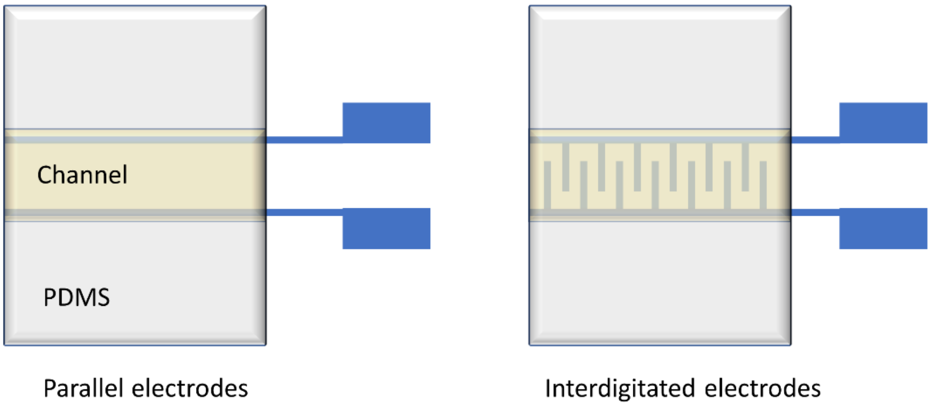

Figure S6: Illustrations of comparisons between microfluidic channels with different widths. By putting the same amount of liquid into each channel, the coverage areas of electrodes for the 'narrower' and the 'same width' channel are similar while this value is smaller in the 'wider' channel. However, the narrower the channels, the faster the liquid reaches the end of the channels, which diminishes its measurement range.

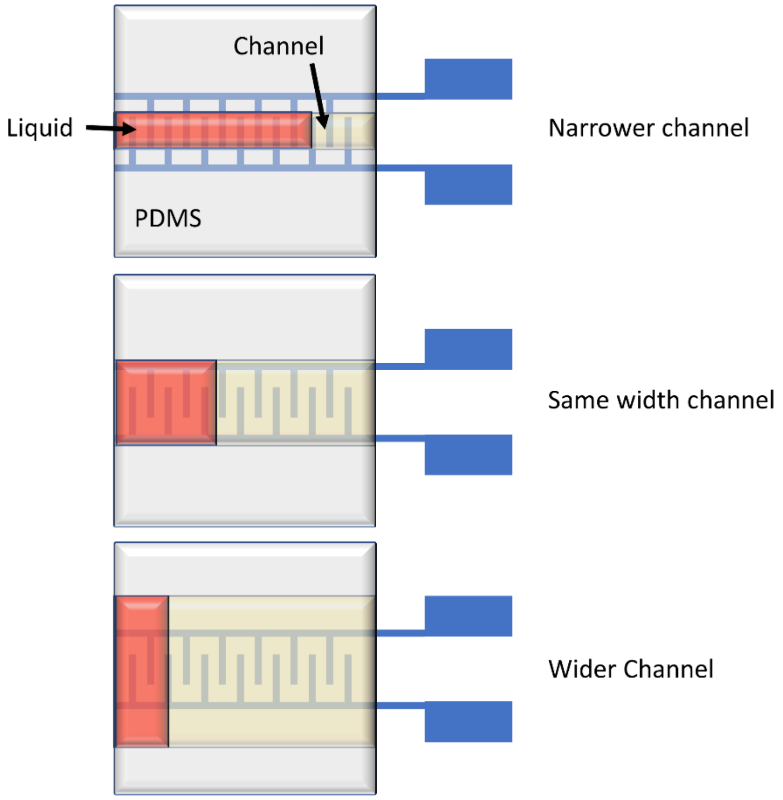

Figure S7: A simulation study of the behaviour of the reservoir under a bending curvature. Results shows a slight effect on the sensitivity of the sensor.

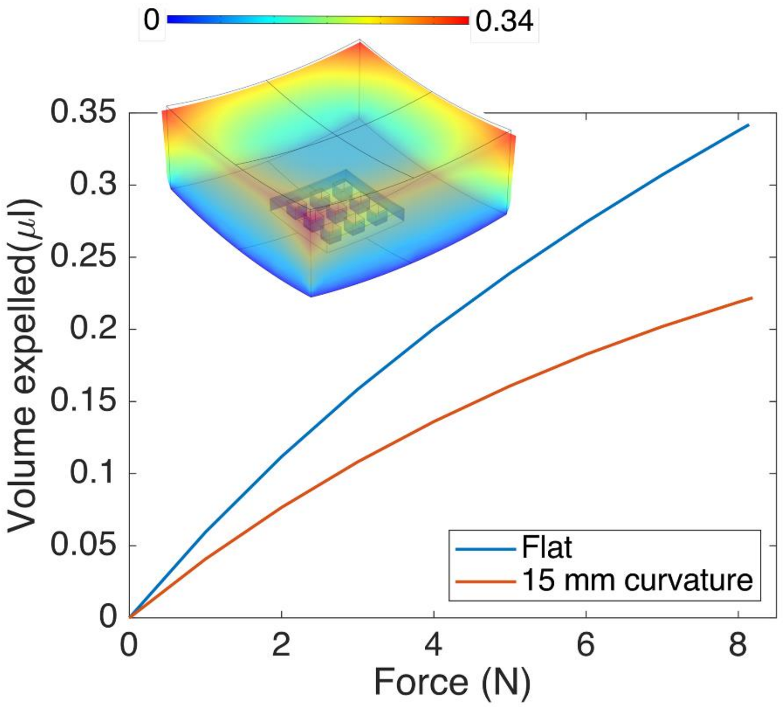

Figure S8: Capacitance values of the force sensors recorded when triggering a robotic clamp. Green dotted lines show the thresholds where, when the capacitance value falls into a certain region, the claws are triggered to act in a defined manner.

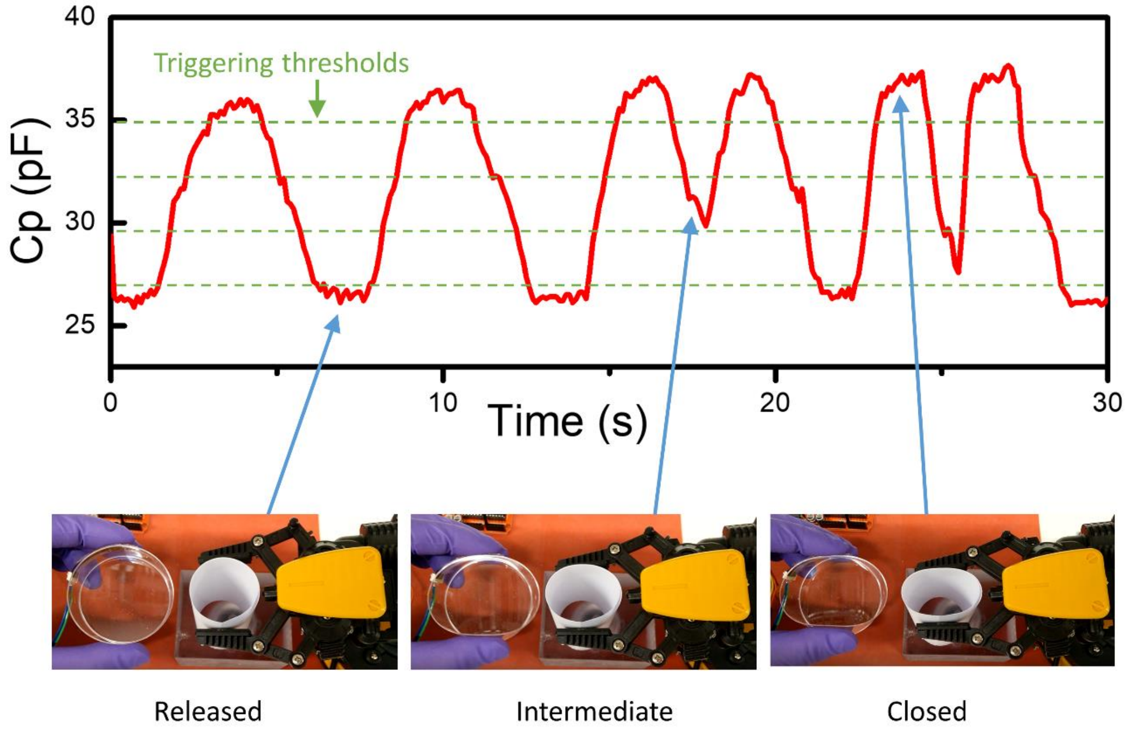

Supplement: Document S1. Figures S1–S8 and Table S1 [file mmc1.pdf]
